# Arginine and Nitric Oxide Pathways in Obesity-Associated Asthma

**DOI:** 10.1155/2013/714595

**Published:** 2013-04-21

**Authors:** Fernando Holguin

**Affiliations:** Division of Pulmonary, Allergy & Critical Care, Asthma Institute, University of Pittsburgh, Pittsburgh, PA 15213, USA

## Abstract

Obesity is a comorbidity that adversely affects asthma severity and control by mechanisms that are not fully understood. This review will discuss evidence supporting a role for nitric oxide (NO) as a potential mechanistic link between obesity and late-onset asthma (>12 years). Several studies have shown that there is an inverse association between increasing body mass index (BMI) and reduced exhaled NO. Newer evidence suggests that a potential explanation for this paradoxical relationship is related to nitric oxide synthase (NOS) uncoupling, which occurs due to an imbalance between L-arginine (NOS substrate) and its endogenous inhibitor, asymmetric di-methyl arginine (ADMA). The review will propose a theoretical framework to understand the relevance of this pathway and how it may differ between early and late-onset obese asthmatics. Finally, the paper will discuss potential new therapeutic approaches, based on these paradigms, for improving the respiratory health of obese subjects with asthma.

## 1. Introduction

Although obesity is associated with less asthma control, greater risk of asthma exacerbations, and reduced inhaled corticosteroid efficacy, whether in fact these conditions are causally related remains uncertain [1]. However, by defining the phenotypical aspects of this relationship, some potential new mechanistic links have been uncovered. Using cluster analyses, obesity has been shown to be an important factor among female patients whose asthma occurs after childhood and have less atopy [2, 3]. This cluster has also been associated with lower airway eosinophils and exhaled nitric oxide (eNO) [3, 4]. The inverse association between body mass index (BMI) and eNO [5] may be explained by an imbalance between L-arginine and one of its methylated products known as asymmetric dimethyl arginine (ADMA) [6, 7]. L-arginine is the substrate that nitric oxide synthase (NOS) uses to generate NO. However, L-arginine is methylated to ADMA, which is an endogenous inhibitor of all NOS isoforms [8]. In addition, ADMA can uncouple NOS and preferentially generate anion superoxide instead of NO [9]. Therefore, conditions favoring a lower L-arginine/ADMA balance would in theory contribute to reducing NO airway bioavailability and increasing airway oxidative stress. This phenomenon has been more extensively studied in the vasculature, as a mechanism leading to endothelial dysfunction or impaired NO-mediated vasodilatation [10]. However, in the lung, the L-arginine/ADMA balance is just beginning to surface as a potential explanation to understand metabolic diseases such as obesity and how these conditions can affect airway function. This review will discuss the role of L-arginine, arginases, and ADMA on airway NO metabolism in relation to obesity and propose a theoretical framework, by which an L-arginine/ADMA imbalance in obesity may explain why obesity leads to worsened respiratory symptoms in some asthmatics. 

## 2. Exhaled Nitric Oxide and Arginine Metabolism; Implications to Obesity and Asthma 

Historically, eNO has been studied as a biomarker of eosinophilic airway inflammation and as a potential biomarker to monitor response to inhaled corticosteroids. Given that high eNO levels track with asthma exacerbations and poor control, it is believed that increased NO could potentially have a causative role in asthma severity, and indeed, this may be the case, if one considers the NO can lead to the formation of more reactive nitrogen species (RNS) in the setting of airway oxidative stress [11]. However, it is unknown whether elevated NO levels per se have any causative role in worsening asthma severity. In fact, NO inhibition lacks clinical benefits in humans, and NO inhalation has been reported to prevent, not to induce, bronchial hyperresponsiveness [12–15]. These results may be explained on the basis that NO, which is constitutively produced by the airway epithelium, contributes to maintaining adequate airway function; for example, NO has been implicated in ciliary beating, maintaining bronchodilation through the formation of s-nitrosothiol compounds, and in bactericidal functions [16, 17]. Also, reduced eNO is associated with several chronic lung diseases [18–20]. Therefore, having reduced eNO in obesity, far from being beneficial, may indicate a major detrimental derangement of NO metabolism [21]. 

Reduced NO bioavailability in obesity and asthma could be potentially explained by several mechanisms, including (a) reduced NOS substrate (L-arginine), (b) endogenous NOS inhibition, (c) combination of (a) and (b), and (d) increased consumption of airway NO into other RNS. Increased arginase expression may reduce L-arginine bioavailability. Arginases are intracellular catabolic enzymes that metabolize arginine into ornithine and urea and have been shown to be higher among patients with asthma [22]. Compared to healthy controls, subjects with asthma have been found to have reduced plasma arginine levels and increased arginase activity, which have been associated with reduced FEV_1_ and greater airway obstruction [23]. Whether obesity could potentially exacerbate this phenomenon is unknown; however, the fact that increased BMI has been associated with increased arginase expression suggests that this might be possible [24]. 

ADMA is one of three methylated analogs of L-arginine occurring through posttranslational modification; however, ADMA is the only one that can competitively inhibit all nitric oxide synthase (NOS) isoforms. ADMA is synthesized from L-arginine by protein-arginine methyltransferases (PRMTs) and degraded into mono- or dimethylamine and citrulline by dimethylarginine dimethylaminohydrolase (DDAH). Conditions such as obesity, metabolic syndrome, and diabetes have been associated with increased PRMT activity and/or reduction in DDAH function. The combination of these enzymatic changes could potentially explain why obesity in asthma contributes to higher ADMA levels [25, 26]. Citrulline can be subsequently recycled into L-arginine [8]. By competing with L-arginine, ADMA uncouples NOS causing electrons flowing from the NADPH reductase domain to the oxygenase domain to be diverted into molecular oxygen rather than to L-arginine [9]. Under uncoupling conditions, NOS generates superoxide, which correlates with airway oxidative stress in murine OVA models [27]. In stimulated murine airway epithelial cells, administration of ADMA reduces nitrite production while increasing superoxide levels in a dose-dependent manner [9]. Continuous ADMA infusion for 2 weeks also increased airway resistance and reduced lung compliance *in vivo* in mice. This increased airway resistance was attributed to reduced NO bioavailability, while the reduced compliance was linked to increased collagen deposition. Interestingly, these findings occurred in the absence of increases in traditional biomarkers of allergic airway inflammation [28].

Evidence that ADMA is associated with reduced eNO in humans is supported by one study showing that sputum ADMA and the L-arginine/ADMA ratio are associated with eNO (*r* = −0.5319 and 0.500, resp., both *P* < 0.05) in adults and children; in addition, sputum ADMA levels were higher in asthmatics versus controls [29]. Plasma ADMA and the L-arginine/ADMA ratio have been found to have similar associations with eNO among late onset asthmatics participating in the Severe Asthma Research Program (SARP) study [7]; however, whether increased airway or plasma ADMA levels have any impact on the respiratory systems is uncertain. Murine OVA models show that pretreatment with ADMA enhances allergic airway inflammation, bronchial hyperresponsiveness, and airway remodeling [9, 28]. These experiments would suggest that increased ADMA plays a role during acute allergic inflammation; yet, the SARP study suggests that reductions in plasma L-arginine/ADMA are associated with reduced FEV_1_, quality of life, and more frequent respiratory symptoms. Based on these results, it could be theorized that lower L-arginine/ADMA balance and its effects on airway NO bioavailability could not only enhance airway inflammation during acute exacerbations but also affect airway function more chronically. 

 Another mechanism potentially contributing to reduced eNO in obesity is airway oxidative stress, which can lead to the formation of RNS and therefore lower the NO fraction that is actually measured [30]. Compared to healthy controls, subjects with asthma have greater concentration of airway oxidative stress biomarkers, which appear to increase in relation to BMI [5, 31, 32]. While this has been demonstrated in exhaled breath condensates and bronchoalveolar lavage, an interaction between asthma and obesity has not been observed in a larger study population using plasma F2-isoprostanes as biomarkers of systemic oxidative stress [33]. It is therefore possible that in subjects with asthma, obesity increases airway and not systemic oxidative stress. The sources for increased airway oxidative stress in relation to BMI are unknown and seem to be (at least at baseline) independent of the number of inflammatory cells present in the airway. Two potential sources may involve a lower L-arginine/ADMA balance leading to the preferential formation of anion superoxide from epithelial NOS and increased airway leptin levels, which have been associated with greater airway levels of proinflammatory cytokines [34]. 

## 3. Arginine and Nitric Oxide Metabolism in Obesity and Asthma, Unique to a Phenotype?

Recent studies have confirmed that asthma is not a single disease but rather a heterogeneous group of clinical entities with different risk factors, varying response to therapies, degree of lung function impairment, and healthcare use, to name a few [35]. While every asthma phenotype has the potential of being obese, the relationship between obesity and asthma may differ. For example, obesity seems to be mostly associated with subjects whose asthma occurs after childhood and have less atopy. This phenotype shows a higher degree of healthcare utilization, with mild lung function impairment and lower eNO [2, 3]. Results from these cluster analyses lead us to hypothesize that the L-arginine/ADMA balance and its relation to NO would differ between the ages of onset asthma phenotypes. Indeed, there were remarkable differences between subjects with childhood (<12 years) versus later (≥12 years) onset asthma. Among later onset asthmatics only, the inverse association between eNO and BMI was partly explained by the L-arginine/ADMA ratio; also, in this phenotype, lower L-arginine/ADMA ratios were associated with poorer asthma-related quality of life, reduced FEV_1_, and more frequent respiratory symptoms, such as wheezing, dyspnea, and chest tightness [7]. Interestingly, there were no associations with cough, sputum production, or increased healthcare utilization. Although this is a cross-sectional study and causation cannot be established, it could be speculated that in this later onset phenotype, lower L-arginine/ADMA leads to reduced airway NO bioavailability, which in turn causes an “airway dysfunction syndrome,” characterized primarily by impaired bronchial dilation without increased sputum production or cough. 

**Figure 1:**
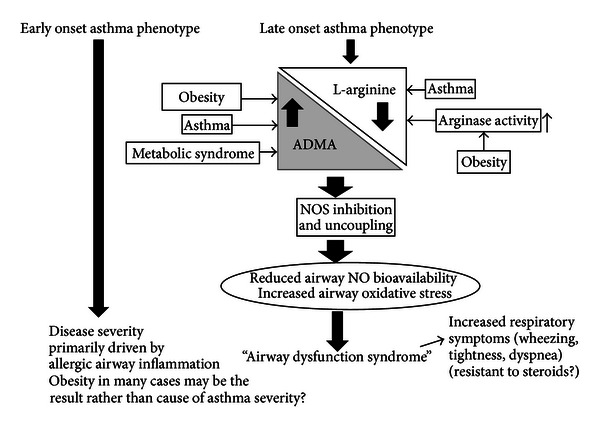
Theoretical framework for the interaction of L-arginine-ADMA and NO in obesity and asthma. Among subjects with late onset (>12 years of age) asthma, reduced L-arginine/ADMA ratios uncouple airway NOS, leading to greater airway oxidative stress and reduced NO bioavailability. In turn, this impairs bronchodilation, worsening wheezing, dyspnea, and chest tightness. Several factors may contribute to having lower L-arginine/ADMA. Obesity and asthma both have been associated with decreased L-arginine bioavailability and increased L-arginine catabolism. Also, obesity has been associated with increased ADMA levels independently from asthma. This algorithm may not be present in those with early onset asthma (<12 years), either because obesity and asthma induce different effects on the L-arginine-ADMA metabolic pathway depending on the age of asthma onset or because other mechanistic pathways drive severity and changes in airway NO metabolism in the early onset asthmatics.

Why there are differences in L-arginine and NO metabolism across age of asthma onset phenotypes is unknown. It is possible that obesity in later onset asthma is associated with changes in L-arginine methylation or arginases expression that are not seen in the early onset phenotype. Alternatively, obesity induces similar changes in both groups; however, in the early onset phenotype other mechanisms (i.e., eosinophilic inflammation, atopy) drive asthma severity and override any effects resulting from the obesity—mediated changes in L-arginine—NO metabolism (see Figure 1).

## 4. Treatment Options

If L-arginine/ADMA is indeed one of the mechanistic pathways by which obesity affects asthma, this could open the door to new therapeutic options. L-arginine can effectively overcome the effects of ADMA on NOS and thus could become an additional treatment for obese late onset asthmatics, particularly for those that have lower or normal eNO and are not highly eosinophilic. Supplementation with L-arginine has been shown to increase eNO in children and adults and to reduce airway inflammation and bronchial hyperresponsiveness in murine ovalbumin sensitization models [36–39]. In addition, L-arginine supplementation can prevent NOS uncoupling [27]. Unfortunately, its use as a therapeutic modality is limited, given its extensive first pass metabolism in the liver and intestine [40]. This is perhaps why one study found only modest improvements in FEV_1_ in asthmatics after 1 week of L-arginine supplementation [41]. Also, because L-arginine catabolism by arginase generates ornithine, a precursor to proline and polyamine, supplementation with L-arginine could theoretically generate collagen synthesis and promote subepithelial fibrosis and airway remodeling. As an alternative, citrulline, a precursor of L-arginine, could be substituted for L-arginine. Citrulline is a nonessential amino acid but essential to detoxify and remove ammonia from muscle and liver cells. It is not subjective to extensive first pass metabolism by gut bacteria or liver arginases and can increase L-arginine levels in a dose-dependent manner [40]. Supplementation with citrulline at a dose of 3 g/BID for 1 week improved the L-arginine/ADMA ratio from 186 ± 8 to 278 ± 14 [40] and rose the levels of L-arginine from 79 (SD ± 8) to 421 ± 65 *μ*mol; in comparison, supplementation with an equivalent L-arginine dose for the same period of time increased L-arginine from 84 ± 9 to 283 ± 51 *μ*mol. These results illustrate that L-citrulline is more effective in increasing arginine plasma levels. 

## 5. Summary

Obesity may lead to abnormalities in the balance of L-arginine and ADMA in obese subjects who acquired asthma beyond childhood. These changes have been associated with increased respiratory symptoms, reduced lung function, and poorer asthma-related quality of life. In addition, murine models support a role for ADMA in enhancing allergic inflammation, oxidative and nitrosative stress. Taken together, these results suggest that the L-arginine/ADMA balance and its effects on NO bioavailability could play a role in obesity-mediated airway disease. These findings are potentially encouraging as they could offer new avenues of treatment—phenotype specific—and are also expanding our ability to characterize asthma beyond quantifying the degree of airway eosinophilic inflammation.
